# Heteroepitaxy of Cerium Oxide Thin Films on Cu(111)

**DOI:** 10.3390/ma8095307

**Published:** 2015-09-18

**Authors:** Josef Mysliveček, Vladimir Matolín, Iva Matolínová

**Affiliations:** Department of Surface and Plasma Science, Charles University in Prague, V Holešovičkách 2, 18000 Prague 8, Czech Republic; E-Mails: josef.myslivecek@mff.cuni.cz (J.M.); imatol@mbox.troja.mff.cuni.cz (I.M.)

**Keywords:** heterogeneous catalysis, monoatomic step, surface oxygen vacancy, oxygen storage capacity, active site, energy conversion and storage, fuel cell, single-atom catalyst

## Abstract

An important part of fundamental research in catalysis is based on theoretical and modeling foundations which are closely connected with studies of single-crystalline catalyst surfaces. These so-called model catalysts are often prepared in the form of epitaxial thin films, and characterized using advanced material characterization techniques. This concept provides the fundamental understanding and the knowledge base needed to tailor the design of new heterogeneous catalysts with improved catalytic properties. The present contribution is devoted to development of a model catalyst system of CeO_2_ (ceria) on the Cu(111) substrate. We propose ways to experimentally characterize and control important parameters of the model catalyst—the coverage of the ceria layer, the influence of the Cu substrate, and the density of surface defects on ceria, particularly the density of step edges and the density and the ordering of the oxygen vacancies. The large spectrum of controlled parameters makes ceria on Cu(111) an interesting alternative to a more common model system ceria on Ru(0001) that has served numerous catalysis studies, mainly as a support for metal clusters.

## 1. Applied and Model Catalysis over Ceria

Despite their name rare earth elements are relatively abundant in Earth’s crust. Particularly elements such as La, Ce, Pr, Nd are as abundant as Cu, Zn, Co or Ni. Due to their ravishing physical and chemical properties rare earth oxides attract broad scientific interest and provide huge amount of applications. An important field of applications is catalysis. Due to its outstanding role in catalysis [1], a substantial amount of publications in the field have been devoted to cerium oxide (CeO_2_, ceria) and many fundamental questions related to ceria surfaces and near-surface processes have been addressed [2]. Considering that the morphology and the electronic structure of ceria strongly influence its chemical and catalytic activity, it is essential to understand in great detail the relations between structure and properties of ceria based systems in order to effectively improve their performance in applications.

Catalysts employing platinum metal (Pt) on a cerium oxide support have received a great deal of attention partly because the nanocomposite Pt-CeO_2_ is of utmost importance for automobile catalytic converters [3,4], which nowadays consume approximately 40% of the worldwide produced Pt-group metals [5]. Pt-CeO_2_ catalysts are also widely used for the water-gas-shift (WGS) reaction, a key step in fuel processing to generate hydrogen [6]. Very recently, novel nanostructured Pt-CeO_2_ material for fuel cell (FC) anode catalysts has been patented by the author [7]. The material is based on nanoporous CeO_2_ thin films prepared by physical deposition techniques, particularly by simultaneous magnetron sputtering of Pt and CeO_2_ [8]. This method permits to prepare oxide layers bulk doped with Pt atoms during the growth and, compared to commonly used chemical wet techniques, it is economical, scalable, and environmentally friendly. For making catalysts, Pt-CeO_2_ thin films can be sputter deposited on various planar or non-planar substrates, including nanoporous Si and C. The Pt-CeO_2_ film shows an exceptionally high activity in mediating formation of protonic hydrogen and it is stable at the anode side of the proton exchange membrane FC (PEMFC) [9,10].

Spectroscopic characterization of this newly developed material of Pt-CeO_2_ revealed a large fraction of the Pt load in cationic Pt^2+^ (on/near the surface) or Pt^4+^ (deeper inside ceria nanostructures) forms [8,9]. This finding strongly indicates that the atomically dispersed Pt cations on nanostructured ceria are essential for the anode FC catalysis rather than metallic particles, so far believed to be essential for that process. Thus, a Pt-CeO_2_ system with extremely low Pt loading—but very high catalytic activity—can be prepared. It is necessary that most of Pt is stabilized as atomically dispersed cations located in surface positions accessible to the reactants during the catalytic process [6,10]. In such a case Pt^n+^-O-Ce interactions not only improve the activity of the Pt-CeO_2_ catalyst but also considerably stabilize the catalyst by making it more resistant to sintering.

The example of Pt-CeO_2_ catalysts for PEMFC illustrates the vast application potential of nanostructured metal-rare earth oxide systems. In spite of the very strong efforts involved in their research, the atomic-level understanding on the local structure and composition of the active catalytic sites in these systems is still lacking or remains incomplete [11]. One central obstacle for reaching this objective is the extraordinary complexity and dynamical character of metal-oxide systems, in general, and those involving rare earth oxides, in particular. Thus, the complexity needs to be reduced by creation and scrutinizing models of the “real” systems that retain most of their key features, but allow a direct assessment of these features by modern research tools [12,13,14,15,16,17]. The research tools for model investigations are not limited nowadays to the standard techniques of surface science, but also very fruitfully include operando experimental studies at realistic pressures of the working atmospheres, and large-scale “computer experiments” carried out using electronic-structure *ab-initio* theoretical methods. For the effective investigation of the structure-property relationships in Pt-ceria and metal-ceria systems the large portfolio of physical methods for highly defined synthesis and atomic-level characterization of nanostructured model ceria, Pt-ceria and metal-ceria systems allows complex model studies of ceria based systems. It includes control of the surface step density [18], the oxygen vacancy concentration and structure [19] and allows complex model studies of ceria based systems shedding light at various physical aspects of catalysis over ceria [10,20,21].

Model studies represent an efficient physical approach based on preparation of well-defined and at atomic level well-characterized surfaces, the simplest case being typically a single crystal, which can be subsequently used for molecule-surface interaction study in conditions of ultra-high vacuum (UHV) [12,13,14,15,16,17]. To obtain a sufficient level of complexity of the model surfaces, and to bridge the so-called materials gap [22] strategies are sought for controlled nanostructuring of model substrates prepared e.g., in the form of oxide *heteroepitaxial thin films* on metals [12,15,17,23].

During last years (metal)-cerium oxide model systems were successfully prepared by growing CeO*_x_* films on Ru(0001) [24,25,26,27,28,29], Pt(111) [30,31,32,33], Au [34] or Cu(111) [35,36] substrates. Further steps toward more realistic modeling of ceria based nanocatalysts require, however, to go beyond the current state-of-the-art and to develop new bottom-up approaches to achieve new degrees of freedom and increased level of control in preparing model ceria surfaces. In parallel it is necessary to develop new advanced techniques for characterization of the electronic and crystallographic structure, charge transfer, the morphology and molecular interactions on nanostructured metal-systems.

## 2. Highly Controlled Ceria Model Catalysts

### 2.1. Continuous CeO_2_(111) Films on the Cu(111) Substrate

**Figure 1:**
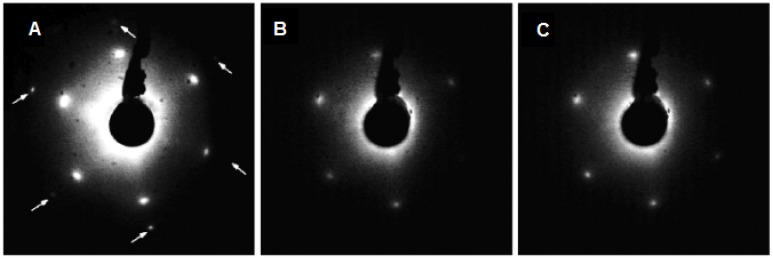
LEED patterns taken at the electron energy of 98 eV for various coverage of CeO_2_ on Cu(111): (**A**) discontinuous film; (**B**) 2.5 ML; (**C**) 5 ML. Weak Cu(111) 1 × 1 spots are marked by the arrows [35]. Copyright Elsevier 2008.

A basic approach to prepare the epitaxial ultra-thin CeO_2_(111) films on the Cu(111) substrate is deposition of Ce metal on clean Cu(111) substrate kept at the temperature of 520 K in a background pressure of 5 × 10^−5^ Pa of O_2_ [35]. This approach yields continuous films of ceria as evidenced by the LEED diffraction patterns in Figure 1 showing no contribution of Cu(111) spots for the films with equivalent thickness above 2.5 ML. Resonance Photoelectron Spectroscopy (RPES, see the Experimental Section) measurements confirm a good CeO_2_ stoichiometry with 5 ML continuous film having predominantly a Ce^4+^ character indicating a negligible concentration of Ce^3+^ surface defects. Discontinuous CeO_2_(111) layers as on Figure 1A exhibit, on the other hand, a higher concentration of Ce^3+^ and defects than continuous layers grown at the same conditions.

The LEED diffraction pattern presented in Figure 1 can be interpreted as the formation of a CeO_2_(111)/Cu(111) epitaxial overlayer with the morphological relationship:

(1) 2*a*CeO_2_ = 3*a*Cu: CeO_2_(111) || Cu(111); CeO_2_[0 1] || Cu[0 1]

where *a* is the surface lattice parameter. The bulk lattice parameter of copper is 0.360 nm, determining the length of the [1 0 −1] lattice vector in the Cu(111) plane *a*_Cu_ = 0.255 nm. The bulk lattice parameter of cubic cerium dioxide is 0.54 nm which corresponds to the [1 0 −1] lattice vector length in the CeO_2_(111) plane of *a*_CeO2_ = 0.382 nm. Thus the expected *a*_CeO2_/*a*_Cu_ ratio is 1.50 indicating very good lattice matching with negligible strain (<0.6%) for the observed (1.5 × 1.5) commensurate superstructure. The absence of rotational domains in the LEED pattern indicates very good ordering of the layer. Epitaxial growth and practically negligible lattice mismatch made the preparation of very thin continuous CeO_2_ film using reactive vapor deposition feasible and it opened a new promising field of model studies of cerium oxide surfaces.

Suitability of the CeO_2_(111)/Cu(111) very thin films for mimicking the cerium oxide single-crystal surface depends on the substrate-oxide interaction that can strongly influence the chemical properties of the ceria/Cu systems as demonstrated in many studies of Cu-ceria inverse catalysts. DFT + U calculations of systems consisting of Cu atoms supported by stoichiometric and reduced CeO_2_(111) surfaces show that Ce^3+^ species are always present underneath the Cu particles supported by stoichiometric and reduced ceria (111) surfaces [37,38]. The calculations predict a substantial charge transfer across the coherent Cu(111)/CeO_2_ interface leading to the full reduction of the first ceria monolayer underneath the supported Cu particles. Therefore the emerging question concerning the physicochemical properties of the CeO_2_(111)/Cu(111) thin films was related to the ceria-copper interaction and the extent to which this interaction determines the properties of ceria/Cu(111). Scanning Tunneling Microscopy (STM) and *ab-initio* calculations allowed to determine the unusual properties of the first ceria monolayer in contact with the Cu(111) substrate showing finite size effects when the limiting thickness of the oxide monolayer and the proximity of the metal substrate cause significant rearrangement of charges and oxygen vacancies compared to thicker and/or bulk ceria [39]. This rearrangement of charges is also responsible for a slight contraction of the lateral lattice constant of ultrathin ceria films on Cu(111) substrate [40].

**Figure 2:**
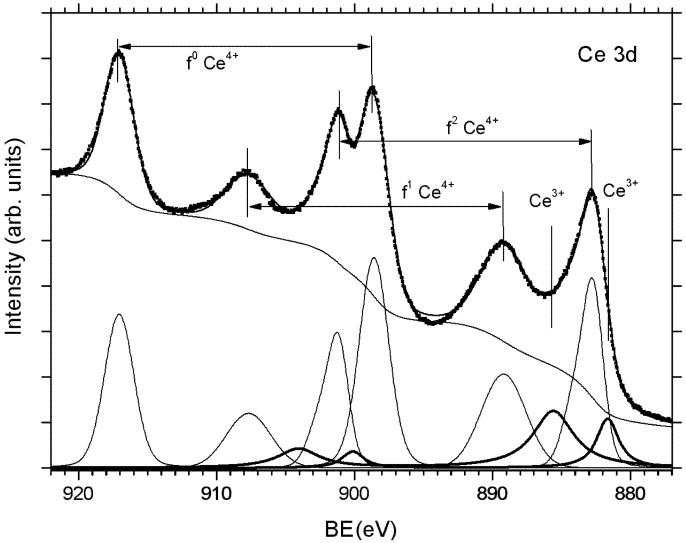
XPS Ce 3d spectrum of partially reduced cerium oxide obtained using Al Kα laboratory X-ray source (1486 eV). Decomposition of the Ce 3d spectrum yields background contribution (monotonously decreasing curve), spectral peak belonging to Ce^4+^ doublets (thin lines) and to Ce^3+^ doublets (thick lines).

The main property of ceria in chemical reactions is the release and the uptake of lattice oxygen to/from the reaction atmosphere. Upon leaving the ceria lattice, the neutral O atom leaves behind two electrons that localize on two Ce atoms occupying the 4f state of Ce [41]. The changes in the occupation of the 4f state result in changes in both valence band spectra and XPS spectra of Ce 3d and Ce 4d core level states due to different final state effects. The stoichiometry of cerium oxide is usually determined by analyzing the Ce 3d XPS spectra. The spectra consist of three 3*d*_3/2_-3*d*_5/2_ spin-orbit-split doublets (*f*^0^, *f*^1^ and *f*^2^) representing different 4*f* configurations in the photoemission final state and arising from 4*f* hybridization in both the initial and the final states [42]. The appearance of a high *f*^0^ signal at 917 eV, together with an *f*^1^ peak (889 eV) which is less intense than the *f*^2^ peak (882.5 eV), is evidence of the formation of CeO_2_ oxide [43,44]. Two spectral components that appear at binding energies BE = 880 and 885 eV correspond to the Ce^3+^ state. In order to estimate the Ce^3+^ state concentration the spectra must be decomposed to elementary doublets. However this is not a simple task because of the ambiguity of background subtraction (the energy interval of the Ce 3d spectrum is too large for correct Shirley background use), choice of elemental peak shape including asymmetry and insufficient spectrometer resolution in general [45]. A typical Ce 3d spectrum of a partially reduced cerium oxide, and the corresponding decomposition of the spectrum into the elementary doublets and the background are shown in Figure 2.

Employing tunable radiation of a soft X-ray synchrotron photoemission beamline, resonance effects in the Ce 4d–4f photoabsorption region can be used to distinguish between Ce^3+^ and Ce^4+^ contributions with very high sensitivity using so called Resonance Photoelectron Spectroscopy (RPES) [44,46].

**Figure 3:**
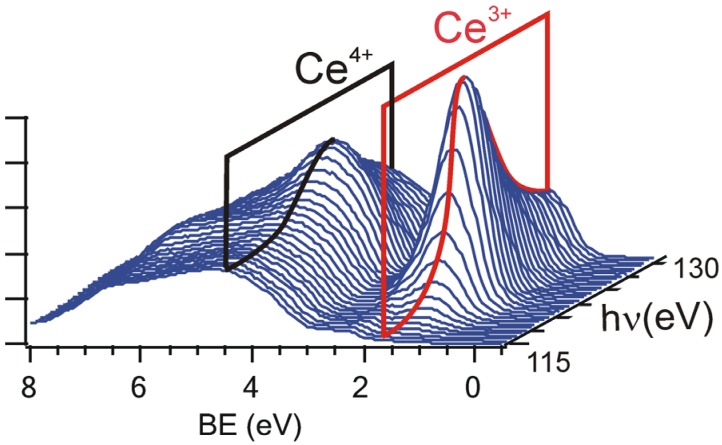
RPES of the cerium 4f level: Ce^3+^ and Ce^4+^ resonant features obtained for the photon energy interval of 115–130 eV. Reproduced with permission from doi:10.1088/0957-4484/20/21/215706, Copyright IOP 2009.

**Figure 4:**
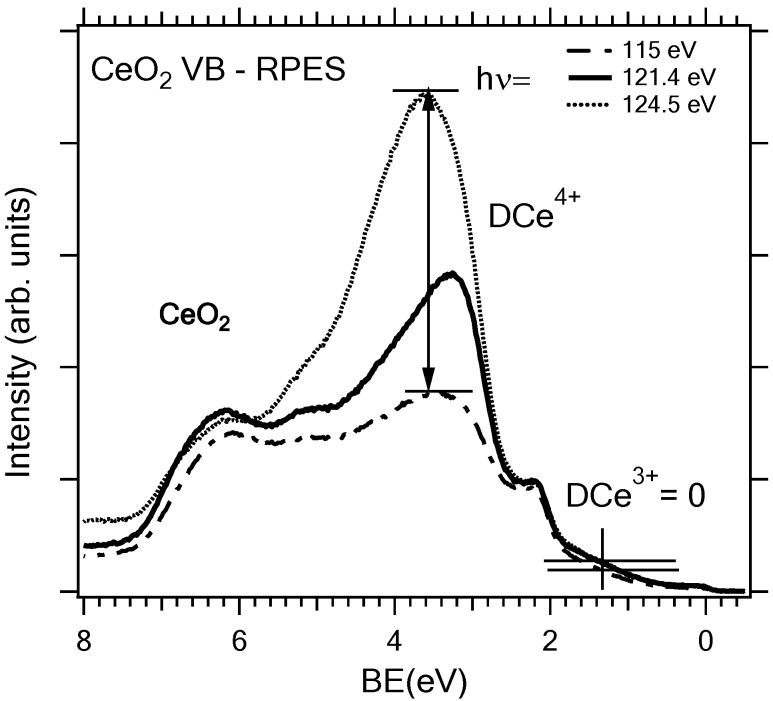
Valence band spectra of the CeO_2_(111) 5 ML thin film on Cu(111) deposited at substrate temperature 523 K taken at photon energies: 115 eV (off-resonance); 121.4 eV (Ce^3+^ resonance); 124.5 (Ce^4+^ resonance). Negligible Ce^3+^ resonance indicates perfect stoichiometry of the model ceria surface.

This method is based on tuning the photon energy in the proximity of the resonant energy where a resonant enhancement of the Ce 4f photoemission can be observed [47]. A series of resonant valence band Ce 4f photoelectron spectra of a partially reduced CeO_x_ is shown in Figure 3 at photon energies hν = 115–130 eV. Two resonances appear for photon energies hν = 121.5 eV and 124.5 eV corresponding to Ce^3+^ (4*f*^1^) and Ce^4+^ (4*f*^0^) valence states. At 115 eV there is no resonance. As we proposed in [35] the density of the 4f states can be obtained by subtracting the off-resonance spectrum from the on-resonance spectrum, *i.e.*, by obtaining so called resonance enhancement DCe^3+^ or DCe^4+^, see Figure 4. The resonant enhancement ratio (RER) DCe^3+^/DCe^4+^ was proposed and is used as a parameter sensitively indicating the degree of reduction of cerium oxide surface. Besides the high sensitivity to small concentrations of Ce^3+^ the energy of detected photoelectrons in the range of 100 eV guarantees the highest surface sensitivity of the RPES the Ce 4d–4f signal.

The resonance spectra shown in Figure 4 were obtained for the sample analyzed by LEED in Figure 1c showing that highly sensitive Ce 4d–4f RPES yields RER close to zero, *i.e.*, perfect stoichiometry of the ceria film.

### 2.2. Adjusting the Morphology of CeO_2_(111) Nanostructured Thin Films on Cu(111)

Scanning Tunneling Microscopy (STM) represents a primary research tool for investigating morphology of nanostructured ceria and metal-ceria samples yielding an indispensable input for the advanced structure-property studies. Local information of the morphology of the model catalysts combines favorably with the information on their electron and chemical state obtained by space-averaging experimental techniques. STM imaging provides information on densities of atomic step edges [18], densities and sizes of metal nanoclusters on ceria [48] or densities of surface oxygen vacancies [20]. Atomically resolved STM imaging provides information on surface reconstructions that in turn represents a complementary information on the charge state of ceria surfaces [39,49].

Adjustable morphology and degree of reduction represent desirable properties of model oxide substrates for heterogeneous catalysis [18] prepared in form of single crystals or heteroepitaxial single-crystalline thin films. The density of atomic steps in the ceria layer determines the dispersion and the electronic structure of the ceria-supported metal clusters [50], because the atomic steps on ceria serve as preferential nucleation sites for many metals [50,51,52,53].

**Figure 5:**
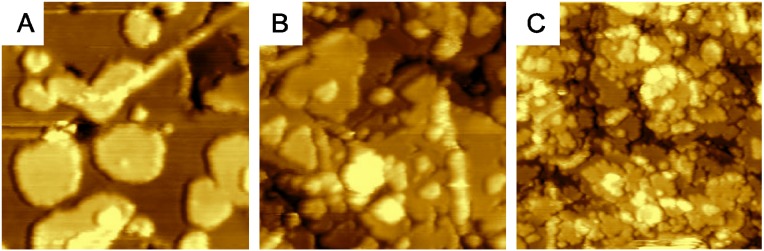
Quantification of morphological features of nanostructured ceria samples by STM. (**A**–**C**) variable density of atomic steps on samples prepared at different temperatures (**A**) 450 °C (**B**) 250 °C (**C**) 150 °C. Image width 60 nm in (**A**–**C**).

A range of bottom-up experimental approaches that allow preparation of oriented thin films of CeO_2_(111) on Cu(111) with deterministically controlled density of atomic steps [18] and the density and spatial ordering of oxygen vacancies [19,49] has been developed. These approaches rely on self-organization properties of cerium and oxygen atoms on the Cu substrate and in ceria and utilize careful control of deposition parameters of the ceria layers. Varying the substrate temperature during layer growth the density of atomic steps can be changed between approximately 5% and 20% (Figure 5A–C) [18].

### 2.3. Adjusting the Stoichiometry of CeO_2_(111) Nanostructured Thin Films on Cu(111)

**Figure 6:**
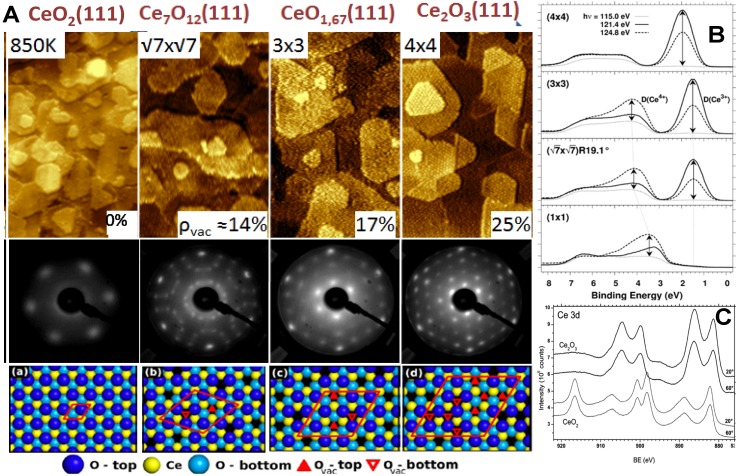
(**A**) STM, LEED and models illustrating the structure of cerium oxide films reduced via interface reaction of metallic Ce with the CeO_2_ buffer layer. (**a**) CeO_2_; (**b**) *ι*-Ce_7_O_12_; (**c**) CeO_1.67_; (**d**) c-Ce_2_O_3_ Reduction of ceria with metallic Ce allows adjusting the concentration of oxygen vacancies between 0% and 25% [19]. Width of STM images 60 nm; (**B**) RPES spectra of the valence band of the ordered phases of stoichiometric and reduced ceria on Cu(111). The spectra are measured off-resonance (photon energy 115 eV, dotted lines), in the Ce^4+^ resonance (124.8 eV, dashed lines), and in the Ce^3+^ resonance (121.4 eV, full lines). The resonance enhancements D (Ce^3+^) and D (Ce^4+^) are indicated by arrows [19]; (**C**) XPS of cerium oxide before (thin lines) and after (thick lines) the reaction between Ce and CeO_2_ buffer layer. The Ce 3d core-level spectra were taken at two different emission angles indicating no changes in the stoichiometry of cerium oxide with information depth [49]*.*

For obtaining a broad range of reduction of ceria layers on Cu(111) we developed a method based on physical vapor deposition of metallic Ce onto a stoichiometric CeO_2_(111) film, *i.e.*, on using metallic Ce as a homotypical reducing species [19,49]. We demonstrated that following the reactive interaction of the two components it is possible to obtain highly ordered films of Ce_2_O_3_ on Cu(111) [19,49] as well as on the Ru(0001) [54] substrate. Ce_2_O_3_ represents the ultimate reduction of ceria that is difficult to obtain by other methods practically used for reducing ceria samples. The extremely sensitive RPES reveals no Ce^4+^ contribution (at 3.56 eV) in the Ce_2_O_3_ layer after reaction (the uppermost spectrum in Figure 6B). The XPS Ce 3d spectra in Figure 6C point that the reaction of ceria layers with metallic Ce yields bulk-reduced samples of Ce_2_O_3_ [49].

Upon stepwise reduction of CeO_2_(111) by metallic Ce, LEED measurements reveal surface reconstructions in the reduced ceria that can be characterized as 1 × 1, (√7 × √7) R19.1°, (3 × 3), and 4 × 4 (Figure 6A), [19]. Photoemission data analysis of the Ce 3d core-level spectra reveals relative concentrations of Ce^3+^ corresponding to surface terminations of ordered bulk phases of reduced ceria, the *ι*-Ce_7_O_12_ or CeO_1.71_ phase for the (√7 × √7) R19.1° reconstruction, the CeO_1.67_ phase for the (3 × 3) reconstruction, and the cubic bixbyite c-Ce_2_O_3_(111) phase for the (4 × 4) reconstruction (Figure 6B). Besides the varying concentration of oxygen vacancies, these bulk reduced phases also represent distinct regular arrangements of oxygen vacancies in cubic ceria.

**Figure 7:**
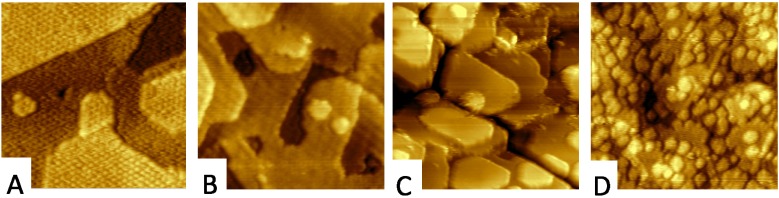
STM micrographs of oxidation of Ce_2_O_3_ layer on Cu(111). (**A**) the starting Ce_2_O_3_ layer; (**B**,**C**) the morphology upon reoxidation; (**C**) reoxidation causes cracking of the ceria layer revealing up to 2% of the Cu substrate; (**D**) upon homoepitaxy of 0.3 ML ceria on the sample from (**B**) at RT and annealing in oxygen at 800 K. Image width (**A**,**B**,**D**) 45 nm; (**C**) 90 nm.

By combining ceria reduction by Ce and oxidation by annealing in oxygen it was shown that both processes are fully reversible. Annealing of the reduced ceria layers in oxygen preserves the morphology of the reduced ceria layer; in particular, the low step density of the Ce_2_O_3_ thin films shown in Figure 7A is preserved upon oxidation to CeO_2_ (Figure 7B) [19]. The CeO_2_ layers obtained by oxidation of Ce_2_O_3_ exhibit the smallest step density and the highest degree of oxidation from the above described model systems CeO_2_/Cu(111). However, the contraction of the lattice constant of ceria upon oxidation causes cracking of the ceria layer revealing up to 2% of the Cu substrate on reoxidized Ce_2_O_3_/Cu(111) samples (Figure 7C). Still, the highly ordered ceria surface represents a suitable substrate for STM experiments that can be further modified e.g., by homoepitaxy and high-temperature annealing of CeO_2_ for increasing the density of steps on the ceria surface without destabilizing the surface thermally (Figure 7D).

Practically, ceria layers reduced by the interface reaction with metallic Ce represent a realization of the ideal scenario of reduction and reoxidation of ceria by removing/adding oxygen from/to the fluorite CeO_2_ lattice without largely modifying the structure of the Ce sub-lattice. This, accompanied by the preference of oxygen vacancies to arrange in regular structures, makes the ceria layers reduced by interface reaction with metallic Ce a unique experimental playground for studying the influence of the oxygen vacancy concentration and coordination on the physico-chemical properties of nanostructured ceria.

## 3. Conclusions

In the present contribution we review methods of preparation and characterization of ceria-based model systems in the form of thin films layers of cerium oxide with different surface stoichiometry, structure, the density of surface steps and oxygen vacancies epitaxially grown on Cu(111).

Heteroepitaxial ultra-thin CeO_2_(111) films of different thickness were grown on the Cu(111) substrate, typically at 520 K in 5 × 10^−5^ Pa of O_2_. The LEED diffraction pattern shows no contribution of Cu(111) spots for the film equivalent thickness above 2.5 ML. The resonance photoelectron spectroscopy at the Ce 3d→4f resonance has been used as an efficient tool for determining the surface stoichiometry of cerium oxide. Discontinuous CeO_2_(111) layers exhibit a higher concentration of defects than continuous layers grown at the same conditions. The 5 ML continuous films can be prepared with practically Ce^3+^ free surfaces; on the other hand concentration of Ce^3+^ site and oxygen vacancies can be tailored by combining growth at constant and variable temperature. We can obtain independent control of coverage and step density of the ceria layers on Cu(111) and prepare ceria layers with adjustable density of the surface steps.

It was shown that interfacial reaction of a stoichiometric CeO_2_ thin film on Cu(111) with deposited metallic Ce yields a highly ordered layer of cubic bixbyite c-Ce_2_O_3_(111). The surface structure of the layer corresponds to bulk-terminated c-Ce_2_O_3_(111). It contains ordered vacancy clusters, each consisting of four oxygen vacancies. The surface exhibits a very characteristic and sharp (4 × 4) LEED pattern relative to CeO_2_(111), allowing easy experimental identification. We suggest that the c-Ce_2_O_3_(111) film is a unique model experimental system for highly reduced ceria surfaces. It provides an atomically well-defined surface exposing exclusively Ce^3+^ ions and a high density of oxygen vacancies with a precisely defined environment. A stepwise reduction of CeO_2_(111) by metallic Ce leads to different surface reconstructions in the reduced ceria that can be characterized as (√7 × √7) R19.1° and (3 × 3) surface structures corresponding to samples with ceria stoichiometry CeO_1.71_ and CeO_1.67_.

The high degree of control of the basic morphological and chemical properties of thin film ceria on Cu(111) presented in this article make them versatile substrates for present and future model catalytic experiments revealing the most important information about the relations between the morphology, stoichiometry, electronic structure and chemical reactivity in ceria based catalysts.
